# Intrinsic Dysregulation and Environmental Modifiers in Hidradenitis Suppurativa: Toward an Integrated Pathophysiologic Model

**DOI:** 10.3390/jcm15166256

**Published:** 2026-08-13

**Authors:** Dorsa Moslehi, Lannika Johnson, Alexandra P. Charrow, Irena Pastar

**Affiliations:** 1 Harvard Medical School, Boston, MA 02115, USA; dorsamoslehi@hms.harvard.edu; 2 Miami Hidradenitis Suppurativa Center, Dr. Phillip Frost Department of Dermatology and Cutaneous Surgery, University of Miami Miller School of Medicine, Miami, FL 33136, USA; laj688@miami.edu; 3 Hidradenitis Suppurativa and Neutrophilic Dermatosis Program, Department of Dermatology, The Brigham and Women’s Hospital, Boston, MA 02115, USA

**Keywords:** hidradenitis suppurativa, pathogenesis, genetics, skin microbiome, androgen signaling, environmental risk factors

## Abstract

Hidradenitis suppurativa (HS) is increasingly recognized as a disorder of intrinsic dysregulation at the intersection of genetic susceptibility, host–microbial interactions, and hormonal signaling, with select environmental exposures acting as important secondary modifiers. This narrative review synthesizes mechanistic, clinical, and epidemiologic evidence on intrinsic and extrinsic contributors to HS pathogenesis. Genetic susceptibility for HS involves pathways regulating keratinocyte differentiation, epidermal stem cell function, and follicular architecture, including Notch signaling and transcriptional regulators such as SOX9 and KLF5. Microbiome alterations in both lesional and non-lesional skin suggest that early dysbiosis may contribute to follicular occlusion, epithelial disruption, and immune activation. Hormonal signaling, particularly androgen signaling, promotes follicular dysfunction and inflammation during periods of hormonal fluctuation, frequently aligning with the time of disease onset and flares. Environmental exposures vary considerably in the strength of supporting evidence: tobacco use and elevated body mass index have the most robust epidemiologic and mechanistic data, heat and humidity are increasingly recognized as disease activity modifiers, and evidence for air pollution and microplastics is growing. Collectively, these findings support a model in which HS arises from intrinsic follicular dysregulation shaped by genetic, microbial, and hormonal factors, with environmental exposures influencing but unlikely to independently initiate disease.

## 1. Introduction

Hidradenitis suppurativa (HS) is a chronic, recurrent inflammatory disease of the follicular unit characterized by painful deep-seated nodules, abscesses, draining tunnels, and scarring, most often affecting intertriginous, apocrine gland-bearing sites, such as the axillary, inframammary, and anogenital regions [1,2,3]. Beyond its cutaneous manifestations, HS is increasingly recognized as a systemic inflammatory disorder associated with substantial pain, impaired quality of life, delayed diagnosis, and frequent comorbidities, including inflammatory arthritis, depression, and cardiovascular risk factors [1,2,3,4].

The pathogenesis of HS remains incompletely defined and multifactorial, with converging evidence implicating genetic susceptibility, follicular epithelial dysfunction, altered keratinocyte differentiation and stem/progenitor cell activity, host–microbial interactions, hormonal signaling, immune dysregulation, and environmental or lifestyle exposures [1,2,4,5,6].

Current evidence supports a model in which genetic susceptibility, host–microbial interactions, and hormonal signaling form a tightly interconnected network, while environmental and lifestyle exposures act on this susceptible background to modify disease onset, distribution, and severity [1,2,4]. Although prior reviews have provided comprehensive overviews of HS epidemiology, clinical burden, immunopathogenesis, and treatment, and recent work has separately examined extrinsic contributors such as mechanical stress, microbiome alterations, smoking, and diet, an important gap remains in integrating these factors into a framework that explains why there is patient variability in disease onset, progression, comorbidity burden, and therapeutic response [1,2,7,8,9,10].

In this review, we synthesize current evidence on intrinsic and extrinsic contributors to HS pathogenesis to address this gap, framing HS as a disorder of intrinsic dysregulation at the intersection of genetics, microbiome, and hormonal signaling, with environmental factors acting as secondary modifiers.

## 2. Methods

A narrative review was conducted using multiple biomedical databases: PubMed/MEDLINE and Embase. Searches combined terms for hidradenitis suppurativa with keywords covering disease pathophysiology, risk factors, genetics, microbiome, hormonal influences, and environmental exposures, including tobacco, obesity, heat, humidity, air pollution, and microplastics. All study designs were considered, and reference lists of included articles were reviewed to identify additional relevant publications. Evidence was synthesized qualitatively, with explicit acknowledgment of evidence quality where relevant.

### 2.1. Intrinsic Factors: A Hallmark of HS Pathogenesis

#### 2.1.1. Genetic Susceptibility to HS

**Figure 1:**
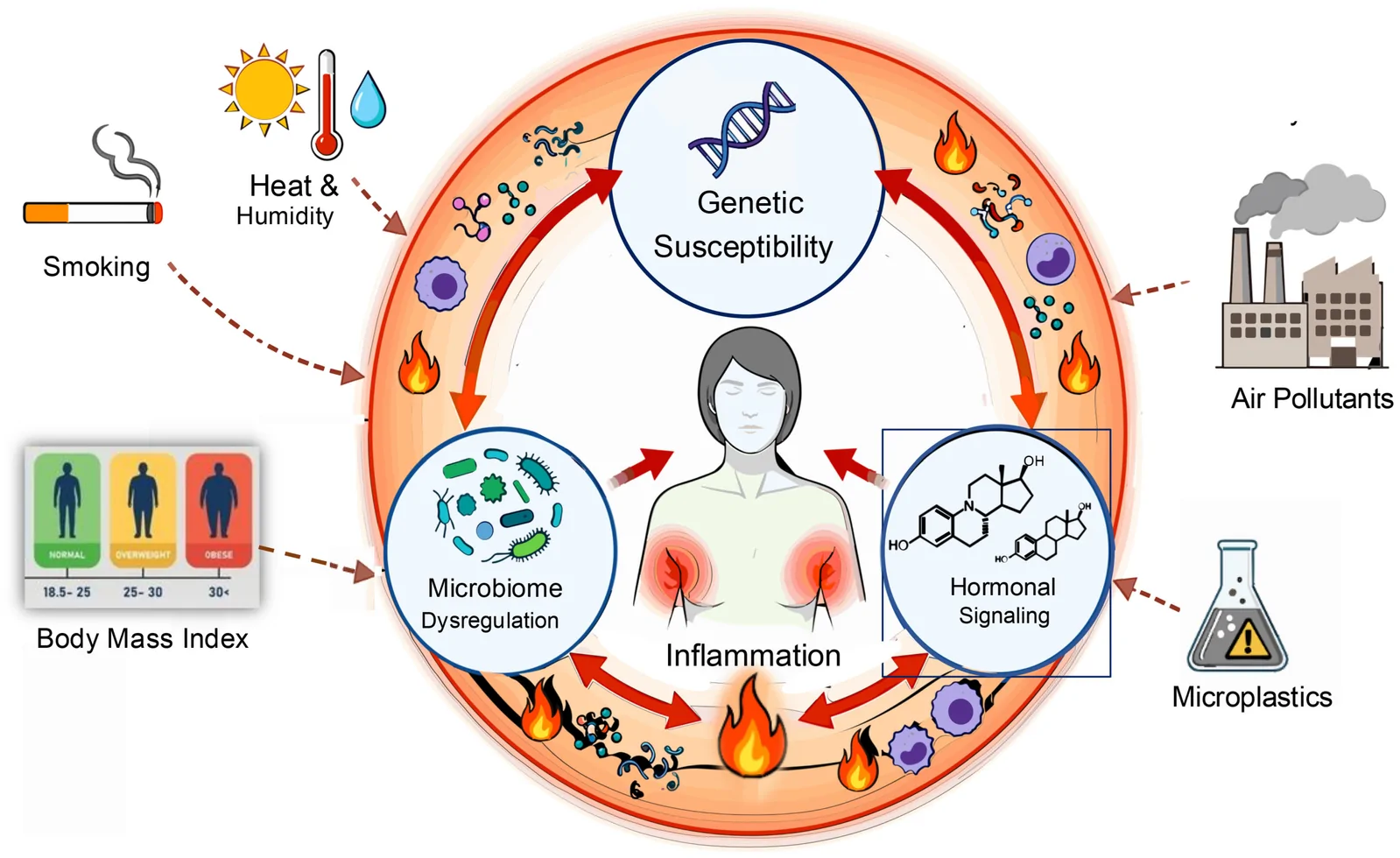
Intrinsic and extrinsic factors contributing to hidradenitis suppurativa pathogenesis. Interconnected intrinsic factors, including genetic susceptibility, microbiome dysregulation, and hormonal signaling, drive follicular dysfunction and chronic inflammation in HS. Environmental and lifestyle factors, including smoking, BMI, heat, humidity, air pollution, and microplastics, act as secondary modulators that amplify or attenuate disease expression through interactions with these intrinsic pathways.

Genetic factors play a significant role in HS. Multiple recent genome-wide association studies (GWAS) have identified HS susceptibility loci that implicate follicular epithelial cells and associated stromal compartments as drivers of disease pathogenesis (Figure 1). HS also demonstrates substantial heritability, exceeding that reported for other well-characterized inflammatory diseases such as psoriasis and rheumatoid arthritis [11,12].

Approximately 30–40% of patients with HS report a positive family history [12,13]. Furthermore, compared to patients without affected relatives, individuals with familial HS tend to develop HS at a younger age and often experience more extensive and longer-standing disease [14]. More recent studies have found that siblings of individuals with HS have an almost 20-fold higher risk of developing the disease [13]. Twin studies from a Dutch cohort of 846 patients estimated HS heritability at 77%, underscoring the strong inherited contribution to disease susceptibility and age of onset [14].

While select HS cohorts demonstrate HLA associations in contemporary GWAS analyses, this immunogenetic region exerts far less influence on HS pathogenesis compared to its dominant role in psoriasis and other characterized immune-mediated inflammatory disorders [15,16,17]. More importantly, early genetic studies identified rare loss-of-function mutations in genes encoding components of the γ-secretase complex, including NCSTN, PSENEN, and PSEN1 [18,19]. This protease complex is required for activation of the Notch signaling pathway, which maintains epidermal homeostasis by promoting keratinocyte differentiation and suppressing proliferation. Disruption of this pathway leads to epidermal hyperplasia, barrier dysfunction, and inflammation—features characteristic of HS [20]. Although mutations in γ-secretase components account for only a minority of HS cases, most commonly observed in Asian populations, these discoveries established an early mechanistic link between disrupted Notch signaling and aggressive, treatment-resistant forms of HS [18]. These findings also helped shift the conceptual framework of HS from a monogenic disorder toward a complex disease involving multiple genetic contributors. Notably, case reports have described new-onset HS following treatment with γ-secretase inhibitors such as nirogacestat, used for desmoid tumors characterized by aberrant Notch pathway activation [21,22,23,24]. The ability of these agents to induce HS in previously unaffected individuals provides compelling human evidence supporting the role of the γ-secretase–Notch axis in keratinocyte homeostasis. These observations further suggest that “acquired” HS may share mechanistic overlap with familial forms driven by loss-of-function mutations in γ-secretase components.

More recent GWAS analyses in larger and more diverse cohorts have identified multiple susceptibility loci independently influencing HS risk. Notably, many lead variants map to regulatory elements active in keratinocytes and dermal fibroblasts rather than classical immune-related pathways [23,25]. Among the most significant loci are regions near the transcription factors SOX9 (SRY-Box Transcription Factor 9) and KLF5 (Krüppel-like factor 5) [26].

SOX9 is highly expressed in the epidermal basal layer and outer root sheath of hair follicles, where it plays a central role in follicular differentiation and stem cell maintenance. GWAS data suggest relative suppression of SOX9 signaling in HS, which may promote follicular degeneration and scarring, keratin dysregulation and fragility, aberrant epidermal differentiation with cyst and tunnel formation, and upregulation of inflammatory mediators [13]. KLF5 is a major regulator of epidermal stem cell function, skin barrier integrity, and lipid metabolism, and genetic polymorphisms predict increased activity of this pathway in HS [26,27]. Additional associations near KLF4 and WNT10A, genes involved in epidermal differentiation and hair follicle development, reinforce the role of disrupted stem cell differentiation and follicular architecture in HS pathogenesis [27,28]. 

Integrative genomic analyses combining epigenomic and transcriptomic data have further refined these findings. Coordinated expression of SOX9, CXCR4, and CD74 has been localized to pathologic epithelial structures in HS tissue, including epithelial tendrils and dermal tunnels, and CXCR4–CD74 signaling has been shown to influence PI3K–AKT and NF-κB pathways, providing a molecular link between epithelial stress, cell proliferation and survival programs, and downstream inflammatory activation in HS [25].

Collectively, these studies indicate that HS genetic susceptibility is enriched in pathways regulating keratinocyte differentiation, epidermal stem cell function, and follicular architecture rather than classical immune pathways alone. Understanding how environmental exposures interact with these underlying defects in follicular biology and skin-barrier function may further elucidate HS pathophysiology and inform strategies for disease prevention and clinical management.

#### 2.1.2. Skin Microbiome as an Understudied Intrinsic Factor in Disease Pathogenesis

The microbiome of intertriginous skin affected by HS differs substantially from that of healthy individuals and from other body sites [29,30]. Next-generation sequencing studies demonstrate that both lesional and non-lesional skin in HS harbor microbial communities distinct from those of healthy controls. The high moisture and sweat secretion, coupled with skin-to-skin contact and mechanical friction, create a unique microbial ecosystem that is different from dry or sebaceous skin and abundant in commensal *Corynebacterium* spp. and *Staphylococcus* spp., fungal *Malassezia*, and smaller proportions of *Streptococcus* spp. and Gram-negative bacterial species [31].

In addition to surface microbiota, the microbiome of the hair follicle lumen is recognized as highly transcriptionally active, maintaining homeostasis in healthy skin by supporting barrier function and modulating immunity [32]. However, the contribution of early hair follicle lumen dysbiosis to HS pathophysiology remains incompletely defined. Within a genetically susceptible host environment, the skin and follicular microbiome likely act as active contributors to disease pathogenesis rather than passive bystanders.

Clinically unaffected skin in patients with HS already demonstrates dysbiotic features, including increased anaerobic bacteria and decreased skin commensals compared with healthy controls, suggesting that microbial imbalance may precede overt lesion formation and contribute to subclinical inflammation [33]. Early-stage HS lesions are characterized by marked reduction in typical skin commensals such as *Staphylococcus epidermidis* and *Cutibacterium acnes* [26,32,33].

Microbial composition then shifts further with disease progression, with disease stage emerging as a major determinant of microbiome structure [34,35]. Early or mild disease (Hurley stages I–II) more frequently contains facultative anaerobes such as *Staphylococcus lugdunensis*, whereas advanced suppurative lesions (Hurley stage III) are enriched with polymicrobial anaerobic communities, including strict anaerobes such as *Prevotella*, *Porphyromonas*, *Peptoniphilus*, anaerobic actinomycetes, and members of the *Streptococcus milleri* group [25,26,32,33]. *Staphylococcus aureus*, the most common skin pathogen, is also frequently isolated from both superficial and deep HS lesions across multiple anatomical sites, including axillary and gluteal regions [36]. Microbial composition may also vary by anatomical site, although findings are inconsistent. Some studies report that groin lesions are more frequently dominated by *Porphyromonas* and *Peptoniphilus*, whereas axillary samples show higher relative abundance of Acinetobacter and the fungus *Moraxella* [29]. Other analyses have found no significant differences between axillary and groin tunnels, instead identifying *Porphyromonas*-dominant dysbiosis as a hallmark microbial feature of HS sinus tracts [37].

Host factors may further contribute to microbial imbalance. Functional reprogramming of apocrine and eccrine sweat glands in HS has been implicated in alterations of the local antimicrobial environment. Transcriptomic studies of HS lesional skin demonstrate downregulation of sweat gland-associated genes, including dermicidin, a sweat gland-derived antimicrobial peptide critical for cutaneous antimicrobial defense. Reduced dermicidin expression may weaken innate antimicrobial activity within intertriginous skin and the hair follicle lumen, increasing susceptibility to microbial dysbiosis [38].

Additional evidence implicates biofilm formation in HS pathophysiology. Biofilms have been described as mucinous bacterial growth within sinus tracts, potentially contributing to chronic inflammation and treatment resistance [35,39,40,41]. Beyond extracellular biofilms, intracellular bacterial reservoirs may also play a role in disease persistence. Recent work has identified *Porphyromonas uenonis*, a rare skin colonizer, as selectively enriched in severe HS. This organism can traverse intact epidermal barriers, persist within keratinocytes, and trigger humoral immune responses while promoting pro-inflammatory keratinocyte signaling [39].

Emerging evidence suggests that the gut microbiome may also be altered in HS patients [40,41,42]. Reported changes include reduced microbial diversity and enrichment of taxa such as *Ruminococcus gnavus*, which may relate to the known association between HS and inflammatory bowel disease [43]. Mendelian randomization analyses further suggest potential causal links between gut microbial composition and HS susceptibility, with taxa such as *Lachnospira* and *Eubacterium* associated with increased risk, whereas members of *Porphyromonadaceae* and Family XI may confer protective effects.

Together, these lines of evidence indicate that the HS microbiome may directly influence epithelial cell behavior and immune activation through microbial metabolites, biofilm formation, and host–microbe signaling pathways, reinforcing chronic inflammation and tissue remodeling [44,45]. Despite accumulating evidence for HS-associated dysbiosis, the interplay between intrinsic host factors and extrinsic environmental influences remains incompletely understood, and current antimicrobial treatment strategies, including topical and systemic antimicrobials, remain largely empirical [46].

#### 2.1.3. The Role of Steroid Hormones in Disease Pathogenesis

Hormonal influences play an important role in HS pathogenesis and may help explain key epidemiologic features of the disease. HS typically develops after puberty and shows a marked female predominance, suggesting that sex hormones contribute to disease susceptibility and activity [44,45].

The timing of disease onset following adrenarche, when terminal hair follicles mature under hormonal control, supports a role for endocrine regulation in disease initiation. Hormonal signaling may interact with underlying genetic susceptibility and immune dysregulation, activating pathways that promote follicular dysfunction and inflammation during periods of hormonal fluctuation, changes that can also alter microbial behavior within the follicular environment [47]. HS frequently begins or worsens during periods of hormonal change such as puberty, menstrual cycles, and pregnancy, and a substantial proportion of women with HS report predictable premenstrual flares, further supporting a hormonal contribution to disease [46,48,49].

These clinical patterns are consistent with the fluctuations in androgen levels during these periods and the biological effects of androgens on follicular physiology. Androgen signaling influences keratinocyte proliferation, sebaceous gland activity, and hair-follicle cycling, processes that can affect follicular occlusion and local microbial composition [50]. Although cyclical hormonal changes are less apparent in men, androgen signaling also appears relevant in male patients. A systematic review of hormones in HS reported increased androgen receptor expression in lesional skin, with sex-specific patterns that suggest androgens play a role in both men and women with HS [47].

Clinical observations further support an androgen-sensitive disease phenotype in a subset of patients. Some individuals develop new-onset HS after initiation of exogenous testosterone therapy, whereas others, particularly male patients, experience improvement with the 5α-reductase inhibitor finasteride [48]. Many female patients with mild-to-moderate disease, particularly those with hormonal triggers such as perimenstrual flares, hyperandrogenism, or features of polycystic ovarian syndrome, experience improvement with spironolactone, which has antiandrogenic activity. Clinical observations suggest reductions in inflammatory lesion burden and pain, while established structural disease (e.g., tunnels, fistulas, scarring) appears less responsive. Overall, these findings suggest that androgen signaling may modulate disease activity in susceptible individuals [41,42].

Emerging evidence indicates that sex hormones may also influence microbial behavior within the follicular environment [51]. Recent experimental studies demonstrate that testosterone can enhance *Staphylococcus aureus* virulence by activating quorum-sensing pathways that increase production of cytotoxic factors capable of promoting tissue damage and impairing immune cell function. Rather than suppressing immunity directly, this hormone-driven increase in bacterial pathogenicity may reduce effective immune clearance and exacerbate inflammation, providing a potential mechanistic link between androgen exposure and disease activity in HS [51].

Taken together, these findings suggest that some patients with HS may exhibit a more androgen-driven disease phenotype, reflecting the combined influence of hormonal signaling, underlying genetic susceptibility, and alterations in the follicular microenvironment [50] This hormonal contribution to disease activity provides a rationale for endocrine-targeted therapies such as androgen receptor antagonists (e.g., spironolactone), combined oral contraceptives, and treatments targeting insulin resistance. Clinical reports suggest that these approaches may improve outcomes in patients whose disease demonstrates a hormonal pattern of activity [47].

### 2.2. Extrinsic Factors Influencing Disease Activity

Marked inter-patient variability is a defining feature of HS and is increasingly recognized as evidence of a dynamic interplay between intrinsic susceptibility and extrinsic modulation rather than a uniform, single-pathway disease process. Rather than being driven by a single dominant factor, HS likely arises from patient-specific interactions between intrinsic programs and extrinsic influences, with variability emerging from differences in how these layers intersect (Table 1, Figure 1). Consequently, disease severity, anatomic distribution, progression, and treatment response reflect not only the presence of risk factors but also the extent to which extrinsic factors reshape underlying intrinsic biology.

#### 2.2.1. Tobacco and Nicotine Exposure as a Risk Factor for HS

Tobacco and nicotine exposure represent the most well-studied and consistently identified environmental risk factors for HS development. While cigarette smoking remains the most extensively studied and historically the most clinically relevant source, other combustible tobacco products (e.g., cigars and hookah), as well as non-combustible nicotine sources such as electronic nicotine delivery systems (e.g., vape pens) and oral nicotine pouches, may also contribute to nicotine exposure.

Epidemiologic studies consistently demonstrate a strong association between tobacco exposure and HS. Up to 80% of patients with HS are current or former smokers [42,52,53]. A comprehensive meta-analysis including 101,977 patients with HS and 17,194,921 controls reported approximately fourfold higher odds of smoking among individuals with HS compared with those without HS [54]. Smoking typically precedes disease onset by a median of 4.5 years in more than 90% of patients [55]. In addition, a retrospective analysis of 50 million patients in the United States found that smoking over a three-year period approximately doubles the incidence of HS among smokers compared with non-smokers [56].

Longitudinal evidence from a large East Asian cohort further supports a temporal relationship between tobacco exposure and HS risk and suggests that smoking cessation may modify this risk. In a large cohort study by Kim et al., more than 6 million individuals were grouped according to smoking status and followed longitudinally until the development of HS, death, or the end of the study period, allowing comparison of HS incidence across smoking categories [57]. Individuals who newly initiated smoking initially exhibited HS incidence rates like never-smokers; however, risk increased after approximately 2–3 years, eventually approaching that observed in sustained smokers. Conversely, individuals who quit smoking maintained HS incidence rates similar to sustained smokers during the first 3–4 years after cessation, after which risk declined and approached that of never-smokers by approximately 3–6 years.

Importantly, individuals who quit smoking had an approximately 33% lower risk of developing HS compared with those who continued smoking. However, this protective effect was delayed, becoming apparent roughly two years after cessation. Participants who relapsed to smoking demonstrated a risk of HS similar to that of individuals who never quit. In sum, sustained smoking cessation for at least three years appears to meaningfully reduce HS risk, whereas continued or resumed smoking maintains elevated risk. These observations highlight smoking cessation as an important primary prevention strategy for HS, while emphasizing that long-term abstinence is required to achieve significant risk reduction [58].

Evidence regarding the effects of smoking among individuals with established HS remains limited, particularly with respect to its impact on disease progression, flaring, and severity. Available studies suggest that active smokers experience greater disease severity, poorer quality of life, and worse treatment outcomes compared with non-smokers [59]. Some data also suggest a dose–response relationship between smoking intensity and disease severity, with severity measured by the number of affected anatomical sites [60].

##### Tobacco and Nicotine Exposure: The Link to Intrinsic Factors Driving HS

Smoking likely contributes to HS development through several interconnected biological mechanisms that converge on intrinsic disease pathways. Many of these effects are mediated by nicotine-induced activation of nicotinic acetylcholine receptors (nAChRs), as well as by other tobacco smoke constituents such as dioxins and polyaromatic hydrocarbons that activate the aryl hydrocarbon receptor (AhR). Nicotine activates nAChRs expressed on keratinocytes, endothelial cells, and immune cells involved in HS pathogenesis. Downstream effects include (1) follicular epithelial remodeling, (2) disruption of the cutaneous microbiome, and (3) amplification of inflammatory signaling pathways [60,61,62,63].

Activation of nAChRs in follicular keratinocytes may promote epithelial thickening, hyperkeratinization, and hair follicle lumen plugging—changes that represent early events in HS lesion formation [64]. Nicotine exposure may preferentially affect the follicular infundibulum, where nAChR expression is highest. Nicotine-mediated cholinergic signaling may also influence the cutaneous microbiome. Nicotine exposure can suppress the expression of antimicrobial peptides in the skin, which reduces the skin’s innate defense against pathogens like *S. aureus* and ultimately increases susceptibility to colonization [65]. *S. aureus* and other skin bacteria can produce or modulate neuroactive compounds, including acetylcholine (ACh)-like molecules, which amplify local cholinergic signaling within the follicular unit and create a feedback loop that sustains microbial dysbiosis and inflammation.

At the immune response level, nicotine-mediated signaling promotes activation of inflammatory pathways implicated in HS pathogenesis, including neutrophil recruitment, macrophage activation, and polarization toward T helper 17 (Th17) responses [53,54]. These processes contribute to amplification of inflammation and sustained immune activation within affected skin. Additional tobacco smoke constituents may further amplify these processes. For example, dioxins and polyaromatic hydrocarbons activate AhR, which promotes pro-inflammatory immune responses that overlap with pathways implicated in HS [66].

#### 2.2.2. Elevated Body Mass Index (BMI) as a Risk Factor for HS

Epidemiologic data consistently demonstrate that higher BMI is associated with increased HS risk and greater disease severity. The relationship between BMI and HS risk appears to begin in childhood. A Danish cohort study of 347,200 children found that HS risk increased with each unit increase in BMI z-score at every age from 7 to 13 years [67]. Children who were overweight at age 13 had approximately twice the risk of developing HS compared with normal-weight peers, and those overweight at both ages 7 and 13 had 2.6 times the risk. Notably, children who were overweight at age 7 but returned to normal weight by age 13 had no measurably increased risk, suggesting that the effect of BMI on HS risk may be reversible. Additional prospective cohort data suggest that each BMI point above 25 is associated with a decreased likelihood of HS remission and that each percentage-point reduction in BMI is associated with a 7% increase in remission likelihood in women [68]. In addition to these observational data, a large Mendelian randomization study of more than 3.5 million participants found that a genetically predicted 1 kg/m^2^ increase in BMI was associated with a 20% increased risk of HS [69].

Evidence regarding the impact of weight loss/BMI reduction on established HS is limited. A retrospective study of patients who underwent bariatric surgery found a point prevalence of HS of 18.1% prior to surgery, compared to the approximately 1% estimated general population prevalence of HS [70]. Among patients who achieved weight loss of more than 15% after surgery, the proportion reporting active HS symptoms decreased by 35%. A 10-year prospective community-based cohort of patients with HS found that among patients who achieved at least 15% weight loss from baseline, all experienced improved disease severity and 80% achieved full remission.

Emerging data suggest a potential role for glucagon-like peptide-1 receptor agonists (GLP-1RAs) in HS management. The largest published multicenter cohort study to date evaluated the use of GLP-1RAs in 66 patients with HS who received GLP-1RAs for at least three months and found that 50–60% of patients achieved clinically meaningful improvement across multiple validated HS outcome measures [71]. However, the extent of weight loss achieved across the cohort was not included, and it is unclear whether the benefit was mediated by weight loss, weight-independent anti-inflammatory effects of GLP-1RAs, or both.

##### Elevated BMI: The Link to Intrinsic Factors Driving HS

At the mechanical level, obesity increases skin-fold surface area, leading to chronic moisture, friction, and relative hypoxia in intertriginous regions. These physical stressors promote microinjury within the follicular epithelium, triggering the release of damage-associated molecular patterns and facilitating microbial entry into the dermis, which can initiate or amplify the perifollicular inflammatory cascade central to HS pathogenesis [1]. Notably, the mechanical impact of obesity on HS development and severity is largely inferred from the characteristic anatomic distribution of HS lesions in intertriginous areas rather than from controlled experimental studies; these mechanical effects, if present, may compound intrinsic follicular abnormalities already present in susceptible individuals.

Adipose tissue expansion can also drive low-grade systemic inflammation overlapping with the inflammatory cytokine profile characteristic of HS. Under conditions of metabolic stress like obesity, hypertrophied adipocytes and adipose tissue-resident immune cells may secrete elevated levels of pro-inflammatory cytokines, including TNF-α, IL-1β, and IL-6, which are among the key mediators driving HS pathogenesis [72,73]. This shared inflammatory environment may lower the threshold for HS development or flaring and sustain chronic inflammation in affected skin.

The occluded, moist, and oxygen-poor microenvironment created by skin folds in patients with obesity can promote overgrowth of anaerobic species that are characteristic of the HS-associated skin microbiome [29]. As such, obesity may shape the cutaneous microbiome in ways that parallel the skin microbiome dysbiosis associated with HS and further amplify local inflammation in the setting of impaired antimicrobial defense, which is intrinsic to HS. Adipokine dysregulation and insulin resistance, both associated with obesity, promote a pro-inflammatory milieu that may reinforce the intrinsic inflammatory programming of the follicular unit [65,66]. However, adipokine dysregulation and insulin resistance are also each independently linked to HS risk and disease activity. Whether these disturbances mediate the effects of obesity on HS or represent independent disease drivers remains to be established.

#### 2.2.3. Climate: Heat and Humidity as a Risk Factor for HS

Heat and humidity are increasingly recognized as contributors to HS disease activity, including in warm climates, during summer months, and in individuals with occupational heat exposure. As global temperatures rise due to climate change, the effects of heat and humidity on disease activity and the associated healthcare burden may increase correspondingly, making these factors increasingly important for clinicians to consider when caring for patients with HS.

Multiple studies have demonstrated seasonal variation in HS flaring, with increased outpatient clinic and emergency department visits during the summer months [67,68]. Building on observations suggesting a relationship between time of year and HS flares, some studies have established an association between higher ambient outdoor temperatures and increased HS disease activity. In a retrospective cohort study examining 2567 patient encounters for HS flares in Boston, between January 2017 and January 2022, investigators identified statistically significant associations between rising ambient temperatures and presentations for HS flares [74]. The strongest correlation coefficient was observed with a 3-day lag between heat exposure and healthcare presentation for flare, suggesting that elevated temperatures may precede increased emergency department utilization by approximately three days.

These epidemiologic findings align with patient-reported experiences. In one survey assessing perceived HS triggers, heat and sweating were the most cited aggravating factors, and approximately 32% of respondents reported worsening of their disease during the summer months [75]. In addition, compared with healthy controls, individuals with HS are more likely to describe sweating as burning, itchy, or painful, suggesting that sweating itself may be perceived as distressing and disease-aggravating, independent of overall HS disease activity [76].

##### Heat and Humidity: The Link to Intrinsic Factors Driving HS

Higher temperatures and humid climates increase sweating as a normal physiologic response; however, the relationship between heat and HS flares does not appear to be explained by increased sweat production alone. Gravimetric studies demonstrate that patients with HS do not exhibit increased sweat production compared with healthy controls, yet multiple reports describe improvement in HS following botulinum toxin A injections, which suppress sweating in affected areas [77,78,79]. Sweat from individuals with HS contains elevated levels of inflammatory mediators compared with healthy controls, and similar mediators are expressed within apocrine glands of HS skin [30]. These findings suggest that sweat may act not only as a physiologic secretion but also as a carrier of inflammatory signals within affected skin.

One potential explanation is that sweating occurs within a cutaneous environment already altered by intrinsic disease processes. As discussed in the intrinsic factors section, functional reprogramming of apocrine glands in HS may increase susceptibility to environmental triggers such as heat and perspiration. In affected individuals, apocrine glands appear to shift from their normal antimicrobial and homeostatic functions toward active participation in inflammatory signaling within the skin.

Follicular hyperkeratosis and plugging, key features of HS pathogenesis, may also obstruct normal sweat drainage, leading to local retention of sweat and associated inflammatory mediators within the follicular unit. Accumulation of these mediators within the follicular environment may promote immune cell recruitment and amplify local inflammatory signaling within the skin [80,81]. At the same time, retained sweat creates moist, occluded, and relatively oxygen-deficient conditions within intertriginous skin folds that promote microinjury and immune activation. These conditions can trigger the release of damage-associated molecular patterns and pathogen-associated molecular patterns, activating resident immune cells and further reinforcing inflammatory pathways involved in HS.

Notably, staphylococcal skin and soft tissue infections demonstrate seasonal variation in the general population, with increased incidence during warmer months, and *Staphylococcus* species are frequently isolated from HS lesions, although their role as primary pathogens or secondary colonizers remains unclear [37,38,39,40]. In the setting of impaired antimicrobial defenses in HS, warm and humid conditions that promote both increased sweating and bacterial proliferation may increase microbial burden within intertriginous skin, further amplifying inflammation and contributing to disease activity.

#### 2.2.4. Emerging Environmental Exposures: Air Pollution and Microplastics as Potential Risk Factors for HS

There is a growing body of scientific inquiry exploring the potential effects of air pollution, microplastics, and their associated endocrine-disrupting chemicals in HS, although current evidence remains largely speculative. The health effects of these environmental exposures have been well studied in other systemic diseases, including more common inflammatory skin conditions, reflecting their substantial public health burden and increasing prevalence in modern environments [82]. However, HS-specific evidence remains limited and consists largely of preclinical or observational data.

Exposure to air pollution occurs in both outdoor and indoor environments. The U.S. government has identified six harmful criteria air pollutants for which National Ambient Air Quality Standards (NAAQS) have been established: carbon monoxide, lead, nitrogen dioxide, ozone, particulate matter (PM2.5 and PM10), and sulfur dioxide. Among these, particulate matter, particularly PM2.5 (particles ≤ 2.5 μm in diameter), has been the focus of most health-related research due to its stronger association with adverse health outcomes [83].

Few studies have examined the relationship between air pollution and inflammatory skin diseases, including atopic dermatitis, psoriasis, and acne. One study demonstrated that air pollution increases the risk of newly developed elderly-onset atopic dermatitis independent of genetic susceptibility, suggesting that environmental exposure may contribute more strongly than genetic variants in this population [84]. Another study used the Google Trends Search Volume Index (SVI), a normalized value ranging from 0 to 100, to examine the relationship between air pollution and itch-related search behavior. The authors found that higher concentrations of PM2.5 were correlated with increased state-level SVI for the term “itch” in 2014 [85]. However, the current state of the literature regarding the role of air pollutants on inflammatory skin disease remains inconclusive, largely due to limitations related to study design. Currently, no large-scale epidemiological studies directly link air pollution exposure to HS. A recent geospatial analysis of Boston-area health system data found significant spatial clustering of HS in south and central Boston and identified neighborhood-level drivers of poor air quality and extreme heat as potential risk factors for HS [86]. Other closely related evidence comes from studies of tobacco smoking in HS, as tobacco smoke represents a major source of indoor air pollution.

Microplastic exposure may occur through several common pathways. Three primary sources relevant to patients include dietary sources, including microplastics present in processed foods; consumer products, such as single-use water bottles and food storage containers; and personal care products, including certain scented, cosmetic, and nail products.

No studies have directly linked measured microplastic burden (e.g., in blood, skin, or stool) to HS risk or disease severity. Existing studies instead rely on indirect behavioral proxies, such as ultra-processed food consumption or plastic use behaviors, which increases the potential for confounding. A cross-sectional study comparing plastic use behaviors among patients with HS and healthy controls found that patients with HS reported more frequent use of plastic-containing products, including greater consumption of canned beverages, more frequent microwaving of food in plastic containers, greater use of single-use plastic water bottles, and fewer home-cooked meals [51]. Another study reported that non-Hispanic Black populations have both the highest consumption of ultra-processed foods and the highest incidence of HS, whereas Hispanic populations consume the lowest amounts of ultra-processed foods and have a lower incidence of HS than non-Hispanic Black populations, despite similar rates of obesity. Because ultra-processed foods are a major source of p-EDCs, the authors proposed that this pattern may suggest a potential relationship between p-EDC exposure and HS risk [87]. However, observed epidemiologic patterns should be interpreted cautiously. Differences in HS incidence across racial and ethnic groups likely reflect a complex interplay of environmental exposures, structural determinants of health, and intrinsic biological factors.

##### Air Pollution and Microplastics: The Link to Intrinsic Factors Driving HS

The mechanisms by which air pollution may influence skin inflammation have been studied primarily in other inflammatory skin diseases, such as atopic dermatitis and psoriasis, and remain largely theoretical in HS. Proposed pathways include activation of the AhR pathway, oxidative stress, disruption of the skin barrier, and inflammasome activation [80].

One biologically plausible mechanism involves AhR-mediated immune signaling. Certain air pollutants, particularly PM, may activate AhR and promote Th17 polarization, potentially exacerbating Th17-driven inflammatory diseases such as HS [66]. This pathway parallels mechanisms proposed for tobacco smoke exposure, in which non-nicotine constituents such as dioxins and polyaromatic hydrocarbons activate AhR signaling. Air pollution may also contribute to skin inflammation through oxidative stress [82]. PM exposure can generate reactive oxygen species (ROS), which damage skin and disrupt lipid components of the skin barrier. ROS can oxidize squalene, a major lipid in sebum, producing squalene peroxides that are highly comedogenic [88]. Because HS pathogenesis begins with follicular occlusion, these oxidized lipids could theoretically worsen follicular plugging and promote local inflammation. However, direct experimental or clinical evidence linking air pollution to HS pathogenesis remains limited.

Like air pollution, the mechanisms linking microplastics or p-EDCs to HS remain largely unproven. Early hypotheses suggested that lipophilic plastic-derived compounds may accumulate systemically and be excreted in sweat, potentially irritating hair follicles and promoting local inflammation [89]. More recent work suggests that p-EDCs may promote inflammatory priming. These compounds have been associated with increased pro-inflammatory signaling and may also interfere with nicastrin, a core component of the γ-secretase complex that regulates Notch signaling and follicular homeostasis [84]. Experimental studies suggest that disruption of this pathway may promote inflammatory responses similar to those observed in familial HS [48]. However, this mechanism has not yet been demonstrated in patients.

**Table 1:** Intrinsic and extrinsic factors contributing to hidradenitis suppurativa pathogenesis, their proposed mechanisms, and implications for clinical care.

Category	Factor	Key Mechanism	Clinical Implications
**Intrinsic**	**Genetic susceptibility**	- Polygenic contributions involving multiple interacting genes, including (1) variants in regulatory elements affecting keratinocyte differentiation and follicular architecture (e.g., SOX9, KLF5) and (2) γ-secretase mutations (e.g., NCSTN, PSENEN, PSEN1) that disrupt Notch signaling - SOX9 plays a central role in follicular differentiation and stem cell maintenance - KLF5 regulates epidermal stem cell function, skin barrier integrity, and lipid metabolism - Notch signaling maintains epidermal homeostasis by promoting keratinocyte differentiation and suppressing proliferation	- Family history may support HS diagnosis and prompt earlier recognition of disease - Consider genetic counseling in patients with severe, early-onset, or familial HS. - Recognition of gene-environment interactions may support early counseling on modifiable risk factors (e.g., smoking cessation) and inform future personalized therapeutic strategies
	**Microbiome dysbiosis**	- Reduction in commensal microbes with a stage-dependent shift toward facultative and polymicrobial anaerobes, biofilm formation, and extracellular and intracellular microbial niches - Promotes epithelial stress and amplifies inflammatory signaling	- Topical clindamycin may provide modest benefit in Hurley stage I-II HS - Oral antibiotics are commonly used for more extensive disease and may provide antimicrobial and immunomodulatory benefit - Antiseptic washes are widely used in practice, though supporting evidence remains limited - Microbiome-directed therapies (e.g., probiotics, bacteriotherapy) remain investigational
	**Hormonal signaling**	- Fluctuations in endogenous and exogenous androgens and increased androgen receptor activity - Affects follicular keratinocyte proliferation, sebaceous gland activity, and hair-follicle cycling - Enhances bacterial pathogenicity, linking hormonal signaling to follicular occlusion, dysbiosis, and inflammation	- Recognition of hormonally influenced HS phenotypes may guide endocrine-targeted therapies - Options include androgen receptor antagonists (e.g., spironolactone), combined oral contraceptives, and therapies targeting insulin resistance [44] - Contraceptive selection may be relevant, as progestin-dominant contraceptives may exacerbate disease activity in some patients [44]
**Extrinsic**	**Tobacco exposure**	- Nicotine-mediated activation of nAChRs expressed on follicular keratinocytes, endothelial cells, and immune cells - Promotes follicular epithelial remodeling, cutaneous dysbiosis, and inflammation - Other tobacco smoke constituents (e.g., dioxins, polyaromatic hydrocarbons) activate AhR signaling, promoting Th17-driven inflammation	- Smoking cessation should be encouraged given strong epidemiologic and mechanistic links between tobacco exposure and HS - Combined behavioral and pharmacologic interventions improve quit rates - FDA-approved pharmacologic options include nicotine replacement therapy, bupropion, and varenicline; longer treatment durations improve sustained cessation [90] - Because nicotine may contribute to HS pathophysiology, nicotine replacement and alternative nicotine products (including vaping) cannot currently be considered risk-free
	**Elevated body mass index**	- Increased skin-fold surface area and associated mechanical effects, including increased moisture, friction, and hypoxia, promote follicular epithelial microinjury and downstream perifollicular inflammation - The skin-fold microenvironment also promotes overgrowth of anaerobic species, leading to skin microbiome dysbiosis - Adipose tissue expansion may contribute to low-grade systemic inflammation overlapping with the HS cytokine profile - Obesity-associated metabolic derangements, including adipokine dysregulation and insulin resistance, promote a pro-inflammatory milieu that may exacerbate intrinsic inflammatory programming of the follicular unit	- Given epidemiologic and Mendelian randomization evidence linking elevated (obese-range) BMI to increased HS risk and severity, weight loss may be recommended for select patients, though evidence that it prevents HS development or improves established disease remains limited and optimal targets have not been established - Among patients achieving ≥15% weight loss, observational data suggest reduced HS activity and improved severity, though controlled trial data are lacking - GLP-1 receptor agonists may benefit select patients, though whether effects are mediated by weight loss, direct anti-inflammatory mechanisms, or both remains unclear
	**Heat & humidity**	- Functional reprogramming of apocrine glands alters sweat composition - Sweat enriched in inflammatory mediators and reduced antimicrobial peptides - Promotes inflammatory signaling and impaired microbial defense - Follicular hyperkeratosis and plugging characteristic of HS further promotes retention of sweat and associated inflammatory mediators within the follicular unit	- Counsel patients on heat and sweat management (e.g., loose-fitting breathable clothing, prompt changing of damp garments, minimizing prolonged heat exposure) - Evidence on antiperspirant use in HS is limited; spray or gel formulations may be better tolerated than solid-stick preparations - Select patients may benefit from sweat-modulating therapies (e.g., botulinum toxin injections), even without hyperhidrosis - May consider seasonal intensification of therapy during warmer months or other periods of increased heat exposure
	**Air pollution ^a^**	- Particulate matter may activate AhR signaling and promote Th17-driven inflammation, paralleling mechanisms described for tobacco smoke exposure via AhR activation - May induce oxidative stress that disrupts skin barrier integrity and amplifies inflammatory responses	- Environmental exposure history may include residential location, occupational exposures, and indoor fuel use - May discuss strategies such as improving ventilation, using indoor air purifiers, and minimizing time in high-pollution environments, though evidence for clinical benefit remains limited
	**Microplastics ^b^**	- p-EDCs may promote pro-inflammatory signaling - May interfere with nicastrin within the γ-secretase complex, disrupting Notch signaling and follicular homeostasis through mechanisms like those implicated in familial HS	- May ask patients about exposures including ultra-processed foods, bottled water use, and plastic food containers, particularly when heating food - May counsel patients to prioritize fresh foods, use glass or stainless-steel containers, and avoid heating food in plastic, though evidence for clinical benefit remains limited

Abbreviations: AhR, aryl hydrocarbon receptor; HS, hidradenitis suppurativa; nAChR, nicotinic acetylcholine receptor; p-EDCs, plastic-associated endocrine-disrupting chemicals; Th17, T helper 17. ^a^ Evidence linking air pollution to HS remains limited. ^b^ Evidence linking microplastics to HS remains limited.

## 3. Conclusions and Future Directions

Emerging evidence supports a pathophysiologic model in which HS arises from intrinsic dysregulation at the level of the hair follicular unit, encompassing genetic susceptibility, host–microbial interactions, and hormonal signaling, with environmental exposures acting as modifiers that can influence disease timing, severity, and heterogeneity.

The strength of evidence for extrinsic factors varies across exposures (Table 1). Tobacco and nicotine exposure are the most well-studied environmental risk factors, with consistent epidemiologic and mechanistic data supporting a causal role. Elevated BMI is also strongly and consistently associated with increased HS risk and disease severity across observational cohort studies and Mendelian randomization analyses. Heat and humidity are increasingly recognized as contributors to disease activity, supported by seasonal variation data and patient-reported flare patterns, though mechanistic evidence remains limited. Evidence for air pollution and microplastics is largely speculative, derived primarily from indirect or preclinical data, and should be interpreted cautiously until larger and more rigorous studies are available.

Whether prolonged extrinsic exposures can become biologically embedded and transition into intrinsic disease drivers over time remains an open question. Current evidence, although limited for some exposures, does not conclusively support this concept. For example, smoking cessation, sustained weight loss, and avoidance of heat and humidity triggers have been associated with reductions in HS risk or disease activity, suggesting that the effects of extrinsic exposures remain at least partially reversible.

However, the absence of definitive clinical evidence does not exclude the possibility that prolonged environmental exposures may induce persistent biological changes. Obesity, tobacco exposure, and diabetes are well-established drivers of epigenetic reprogramming, while GWAS risk variants for HS are predominantly located within non-coding regulatory regions of the genome [13,91,92,93]. Together, these observations provide a biologically plausible framework whereby environmentally induced epigenetic alterations could influence disease-relevant pathways, including keratinocyte differentiation, stem cell function, and host responses to the microbiome, thereby disrupting follicular homeostasis, promoting immune activation, and ultimately contributing to disease progression [4,87]. Whether these mechanisms causally mediate long-term disease persistence in HS, however, remains to be established.

Future research should better characterize the interplay between environmental exposures and intrinsic factors, including how these interactions influence the timing, severity, and heterogeneity of HS disease expression and whether cumulative environmental exposure can induce durable biological and epigenetic changes. Longitudinal studies integrating genomic, epigenetic, microbiome, hormonal, and environmental exposure data will be important for identifying causal pathways and defining clinically meaningful disease subtypes, while paving a path towards personalized treatment strategies.

## Data Availability

No new data were created or analyzed in this study. Data sharing is not applicable to this article.
